# The development of new Electronic Health Record Exchange Format Use Cases - an evaluating perspective: Anja Stradner

**DOI:** 10.1093/eurpub/ckac131.171

**Published:** 2022-10-25

**Authors:** K Trunner, E Leuprecht, A Stradner, C Pinto, S Franconi, M Melgara, A Boumpaki, F Soares, G Liapi

**Affiliations:** 1 International Affairs and Consultancy, Austrian National Public Health Institute GÖG, Vienna, Austria; 2 International Projects and Affairs, Shared Services of the Ministry of Health, Lisbon, Portugal; 3 European Affairs, Integrating the Healthcare Enterprise-Europe, Brussels, Belgium; 4 International Research Projects, Innovation & Procurement Regional Company Lombardy, Milan, Italy; 5 Electronic Health Services Department, Hellenic Ministry of Health, Athens, Greece; 6 Department of Computer Science, University of Cyprus, Nicosia, Cyprus

## Abstract

The EU-funded X-eHealth project aims to lay the foundations for a common framework for medical imaging, discharge letters, laboratory results and rare diseases to advance an interoperable Common European Health Data Space for citizens and healthcare providers in accordance with privacy and cybersecurity regulations. To ensure sustainability of the project, it is crucial to assess whether X-eHealth is achieving its planned objectives and delivers tangible results. A key challenge in evaluating project outcomes lies in the partial lack of visibility of direct impacts due to a time lag of effects or attribution problems (i.e., identifying the main cause of a particular impact). In X-eHealth, we address this problem by defining a framework of Key Performance Indicators (KPI) that allows the monitoring of project outcomes. In addition, qualitative interviews with a focus on the following questions are conducted towards the end of the project to complement the evaluation:

• Does X-eHealth advance the integration process of eHealth services in Europe?

• Will X-eHealth lead to increased use of the new European Electronic Health Record Exchange Format (EEHRxF) specifications?

Although final results are not yet available, we expect X-eHealth to have a positive impact at all stakeholder levels by accelerating the implementation of EEHRxF through the harmonisation of health data, thus providing patients, health professionals and institutions with increased quality, safety and efficiency. There is little evidence to analyse the impact of eHealth policy interventions. For this reason, the present evaluation makes a significant contribution to impact research in this area. X-eHealth highlights the importance of international cooperation in creating a common framework for the future development of digital health in the EU. The continuous evaluation, moreover, facilitates a more efficient management of resources through KPI observance and the implementation of project results.

**Key messages:**

• X-eHealth aims to accelerate the implementation of the European eHealth Record exchange Format (EEHRxF) through the standardisation and harmonisation of health data.

• By evaluating and monitoring the project processes, it is possible to ensure the progress of the project and to measure the potential impacts of the outcomes on stakeholders and other target groups.

